# Effects of sleep deprivation on cognitive and physical performance in university students

**DOI:** 10.1007/s41105-017-0099-5

**Published:** 2017-04-13

**Authors:** Yusuf Patrick, Alice Lee, Oishik Raha, Kavya Pillai, Shubham Gupta, Sonika Sethi, Felicite Mukeshimana, Lothaire Gerard, Mohammad U. Moghal, Sohag N. Saleh, Susan F. Smith, Mary J. Morrell, James Moss

**Affiliations:** 1 Imperial College School of Medicine, Imperial College London, South Kensington Campus, Sir Alexander Fleming Building, London, SW7 2DD UK; 2 Academic Unit of Sleep and Breathing, National Heart and Lung Institute, Imperial College London, London, UK; 3 NIHR Respiratory Disease Biomedical Research Unit, Sleep and Ventilation, Royal Brompton and Harefield NHS Foundation Trust, Sydney Street, London, SW3 6NP UK; 4 Faculty of Medicine, Imperial College London, South Kensington Campus, Sir Alexander Fleming Building, London, SW7 2DD UK; 5 Medical Education Research Unit, Faculty of Medicine, Imperial College London, South Kensington Campus, Sir Alexander Fleming Building, London, SW7 2DD UK

**Keywords:** Student, Acute sleep deprivation, Reaction time, Cognitive, Submaximal exercise

## Abstract

Sleep deprivation is common among university students, and has been associated with poor academic performance and physical dysfunction. However, current literature has a narrow focus in regard to domains tested, this study aimed to investigate the effects of a night of sleep deprivation on cognitive and physical performance in students. A randomized controlled crossover study was carried out with 64 participants [58% male (*n* = 37); 22 ± 4 years old (mean ± SD)]. Participants were randomized into two conditions: normal sleep or one night sleep deprivation. Sleep deprivation was monitored using an online time-stamped questionnaire at 45 min intervals, completed in the participants’ homes. The outcomes were cognitive: working memory (Simon game© derivative), executive function (Stroop test); and physical: reaction time (ruler drop testing), lung function (spirometry), rate of perceived exertion, heart rate, and blood pressure during submaximal cardiopulmonary exercise testing. Data were analysed using paired two-tailed *T* tests and MANOVA. Reaction time and systolic blood pressure post-exercise were significantly increased following sleep deprivation (mean ± SD change: reaction time: 0.15 ± 0.04 s, *p* = 0.003; systolic BP: 6 ± 17 mmHg, *p* = 0.012). No significant differences were found in other variables. Reaction time and vascular response to exercise were significantly affected by sleep deprivation in university students, whilst other cognitive and cardiopulmonary measures showed no significant changes. These findings indicate that acute sleep deprivation can have an impact on physical but not cognitive ability in young healthy university students. Further research is needed to identify mechanisms of change and the impact of longer term sleep deprivation in this population.

## Introduction

Sleep deprivation is common amongst university students whom live in a culture that promotes reduced sleep, due to the burden of academic work and social pursuits. The reasons for poor sleep hygiene include alcohol and caffeine intake, stimulants, and technology, which prevent students achieving sufficient sleep time and quality [1]. A cross-sectional survey found that 71% of students did not achieve the recommended 8 h of sleep, with 60% classified as poor sleepers [2]. An average of 5.7 h sleep has been reported for students studying architecture, and sleepless nights due to academic work throughout the night—defined by the Oxford English Dictionary as an all-nighter—occurred, on average, 2.7 days a month [3].

While many studies have investigated the effects of acute sleep deprivation, few focus on university students, despite the prevalence and impact of sleep deprivation in this population [4, 5]. Such studies often have a narrow focus on disease states, limiting their ability to provide a holistic assessment of physical, emotional and cognitive wellbeing [4–6]. The importance of physical and cognitive function is especially appreciable in the student population, 52% of whom play sport at least once a week. Moreover, students rate sleep problems second only to stress in relation to negative impact on academic performance [7]. The effect of acute sleep deprivation on physical performance has been well documented with negligible effects on intense periods of exercise, whilst endurance task performance suffers due to decreased motivation [8, 9].

The effect of sleep deprivation on cognitive performance has also been documented previously with a correlation between sleep quality and grade point average in first year university students [10]. Moreover, sleep deprivation has been shown to have a detrimental effect on certain aspects of working memory, such as filtering efficiency, whilst Stroop test scores show degradation; however, this has been evidenced to be due to deficits in reaction time rather than processing skills [5, 11–17]. Taken together, these data suggest that sleep deprivation may have a limited effect on cognitive ability in university students.

This study aimed to determine whether a night of sleep deprivation, equivalent to an “all-nighter”, would have a negative impact on the motor and cognitive performance of students, specifically focusing on reaction time, executive function, working memory, and cardiopulmonary function.

## Materials and methods

### Study design, participants, and recruitment

This was a randomized, controlled crossover study, which took place from June to September 2015. Exclusion criteria were: (1) any medication or medical history that would make participation in the study, in particular the sleep deprivation and exercise test, unsafe, or inappropriate; (2) mental incapacity to provide informed consent, or (3) recent (within 6 months) participation in a research trial. Participants were recruited via direct approach and posters on campus, social media, and a National Heart and Lung Institute newsletter. Participants travel expenses were reimbursed and all participants were offered the opportunity to be entered into a prize draw. Participants were told that the study involved testing parameters following sleep deprivation, but no information was given regarding the anticipated results. All participants gave written informed consent, and the study was approved by Medical Education Ethics Committee (Imperial College London, 23/4/15, MEEC1415-24).

Participants were randomized to either the sleep deprivation or a normal night’s sleep first, using a random number sequence. Twenty-four hours prior to the morning assessment, participants were instructed to refrain from consuming alcohol and caffeinated drinks as well as abstaining from exercise, smoking, and nicotine patches. Those having a normal night’s sleep where asked to report how much they had slept. The sleep deprivation arm were required to fill out a form every 45 min to confirm that they were still awake. This form was checked the following morning. More than two unexplained missed form completions resulted in disqualification from the study. The crossover condition and assessment were undertaken within 3–12 days of one another. Testing occurred between 09:00 to 13:00, with participants being allowed flexible timings; however, all follow-up testing aimed to take place within 1 h of initial session time. The outcome was to measure the change, if any, which occurred between the cognitive and physical performance of participants undergoing sleep deprivation.

Before testing began, height and weight were recorded and participants were asked to fill out a fitness questionnaire. The results of this questionnaire and participant sex were used to estimate the appropriate Monark Ergomedic 828e resistance for each participant, (male: 2.0 kp = unfit, 2.5 kp = fit, 3.0 kp = athlete; female: 1.5 kp = unfit, 2.0 kp = fit, 2.5 kp = athlete). A second questionnaire enquired about recent (within 24 h) intake of food, caffeine, alcohol, and nicotine, and any physical exercise was also completed.

All participants were provided with standardized descriptions of tests and given the opportunity to habituate with procedures.

### Cognitive function tests

The working memory mobile application was derived from the SIMON© game, an appropriate test for working memory span [18]. It involved repeating a random sequence of colors and sounds. As each level progressed, another random color-sound combination was added to the previous sequence. This test was repeated three times.

Standard stroop charts were used: (1) monochrome (reading black text); (2) conflicting color (reading words with a mismatched color); (3) color blocks (articulating the color of colored blocks), and (4) conflicting words (articulating the color of mismatched words) [19]. Four versions of each test were created, so that no participant used the same chart twice. Time taken to complete each chart and the number of mistakes were recorded providing a measure of selective attention, automatic responses, inhibition, and control of executive functions [20–22].

### Physical function tests

Participants performed two concordant volume-time spirometry traces, in adherence to standard guidelines [23].

**Fig. 1:**
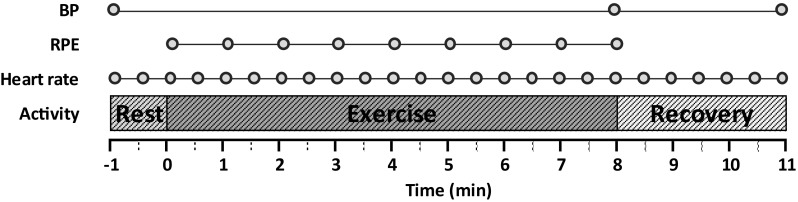
Timeline showing the measurements taken during the CPET. After calibration, a one-minute baseline was conducted followed by 8 min of exercise and 3 min of rest. Non-invasive blood pressure (BP) measurements were taken using a manual sphygmomanometer at three points of the test: pre-exercise, post-exercise, and post-rest period. Rating of perceived exertion (RPE) (53) was recorded at the end of each minute

Participants underwent submaximal 8 min cardiopulmonary exercise testing (CPET) using a cycle ergometer target cadence 50 ± 5; this intensity of exercise test was chosen to replicate more closely students’ daily activities (as opposed to maximal exercise testing). Three electrocardiogram (ECG) electrodes were attached, and non-invasive blood pressure (BP) measurements and rating of perceived exertion (RPE) were also recorded throughout the exercise test (Fig. 1). Measurement of RPE has repeatedly been shown to have a strong correlation with the intensity of exercise being performed, independently from other factors [24–26].

### Reaction time

The ruler drop test was used to assess average reaction speed, a simple and inexpensive test compared to computerized assessments, with comparable reliability [27–30]. Participants carried out three practice runs to eliminate a learning effect [27].

### Statistical analyses

Statistical tests were carried out using IBM SPSS Statistics (V 22.0), Armonk, NY, USA. Due to the novel measures used in this study, a preliminary study was performed to estimate appropriate sample size. The study consisted of eight participants representative of the target population with a target sample size determined of 51 at a power level of 80%; therefore, a target recruitment of 70 was set with an estimated 25% attrition rate. Initially, descriptive statistics were obtained for all variables. Data were inspected for normality using histograms. Parametric data were reported as mean ± standard deviation, and paired two-tailed *T* tests were carried out to assess the difference between normal night sleep and sleep deprived arms. The tests were carried out with alpha significance level *p* ≤ 0.05, and 95% confidence intervals were calculated. Levene’s test (homogeneity of variances) was used to assess the differences in variance of samples. Differences in rating of perceived exertion and heart rate during exercise were assessed using multivariate analysis of variance test.

## Results

### Participant characteristics

**Fig. 2:**
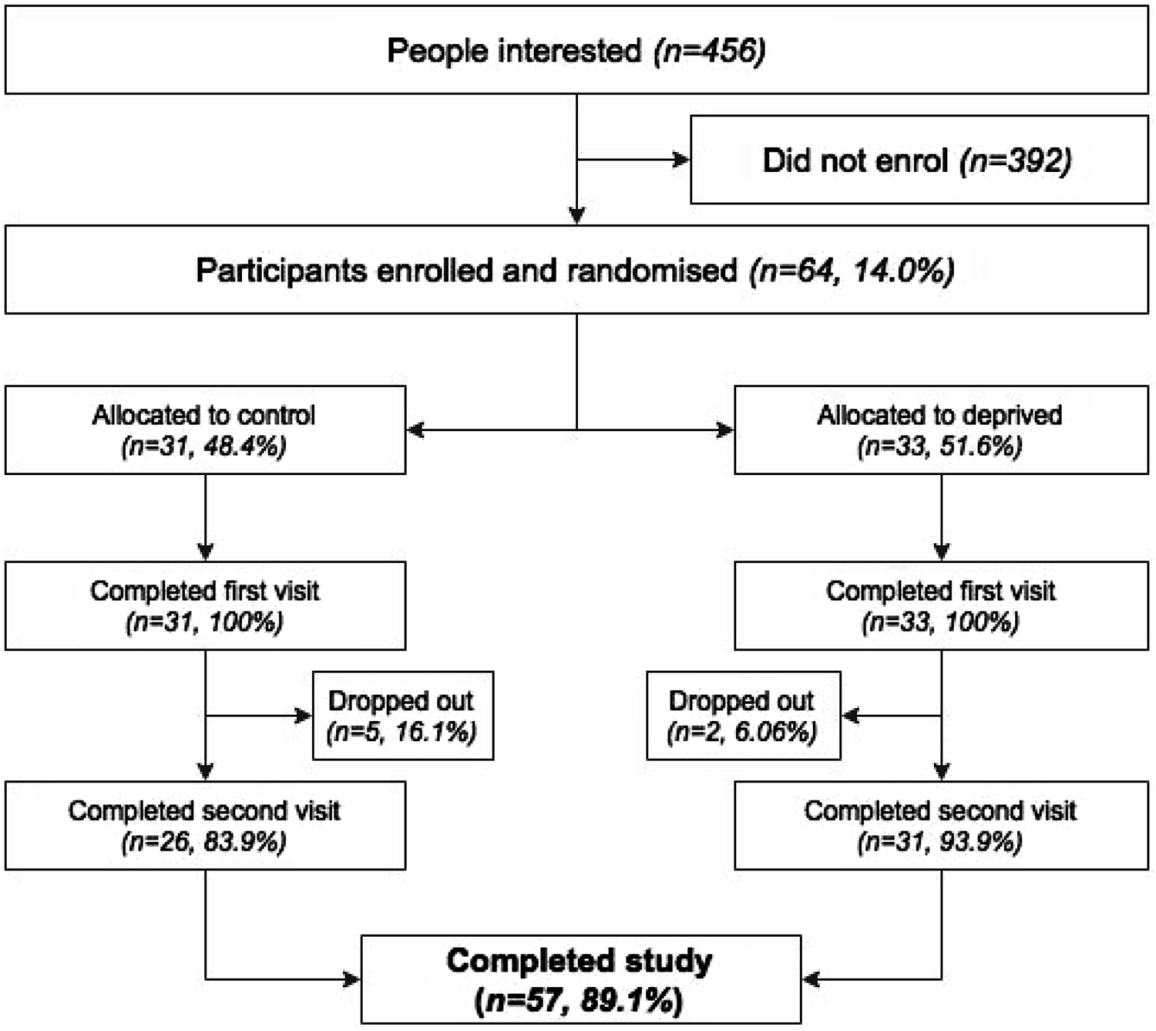
Flow chart showing participant numbers during the study. *Percentages* indicate the percentage of individuals who remained from the previous stage

The study sample consisted of 64 Imperial College London students, 57 (89%) of which completed the study (Fig. 2). The characteristics of the participants are summarised in Table 1; upon study entry, participants reported typically sleeping between 5 and 9 h per night, with 94.7% reporting these hours as ‘typical’ and 98.2% reporting sleeping through until morning without waking on most nights. 58% of participants were male and 69% were undergraduates. The participants’ mean age was 22 ± 4 years. Participants in the sleep deprivation arm filled in the online form on average every 49 ± 21 min throughout the night. Participants in the normal night’s sleep arm reported sleeping on average 7.2 ± 1.0 h. The mean difference in time of day that testing occurred (between condition 1 and condition 2) was 32 ± 15 min with no significant difference between testing times (*p* = 0.220).

**Table 1:** Participant characteristics (*n* = 57)

Characteristic	Normal night sleep (*n* = 57)	Sleep deprived (*n* = 57)
Age (years)	22 ± 4	
Weight (kg)	67 ± 14	
Height (m)	1.7 ± 0	
Body mass index (kg/m^2^)	23 ± 4	
Normal sleep duration (h)	7.4 ± 1.0	
Disrupted normal sleep [*n* (%)]	1 (1.8%)	
Sleep-related characteristics		
Quality of sleep (/10)	7.48 ± 1.99	0.67 ± 1.64
Current mood (/10)	7.21 ± 1.69	3.51 ± 2.42
Rating of adequacy of sleep (/10)	7.40 ± 2.02	0.69 ± 1.73

Data are reported as mean ± standard deviation. Sleep-related characteristics were obtained using a questionnaire with a 10-point scale, ranging from 0 (lowest quality sleep, poor mood, and inadequate sleep) to 10 (best quality sleep, good mood, and fully adequate sleep)

### Exercise testing

**Fig. 3:**
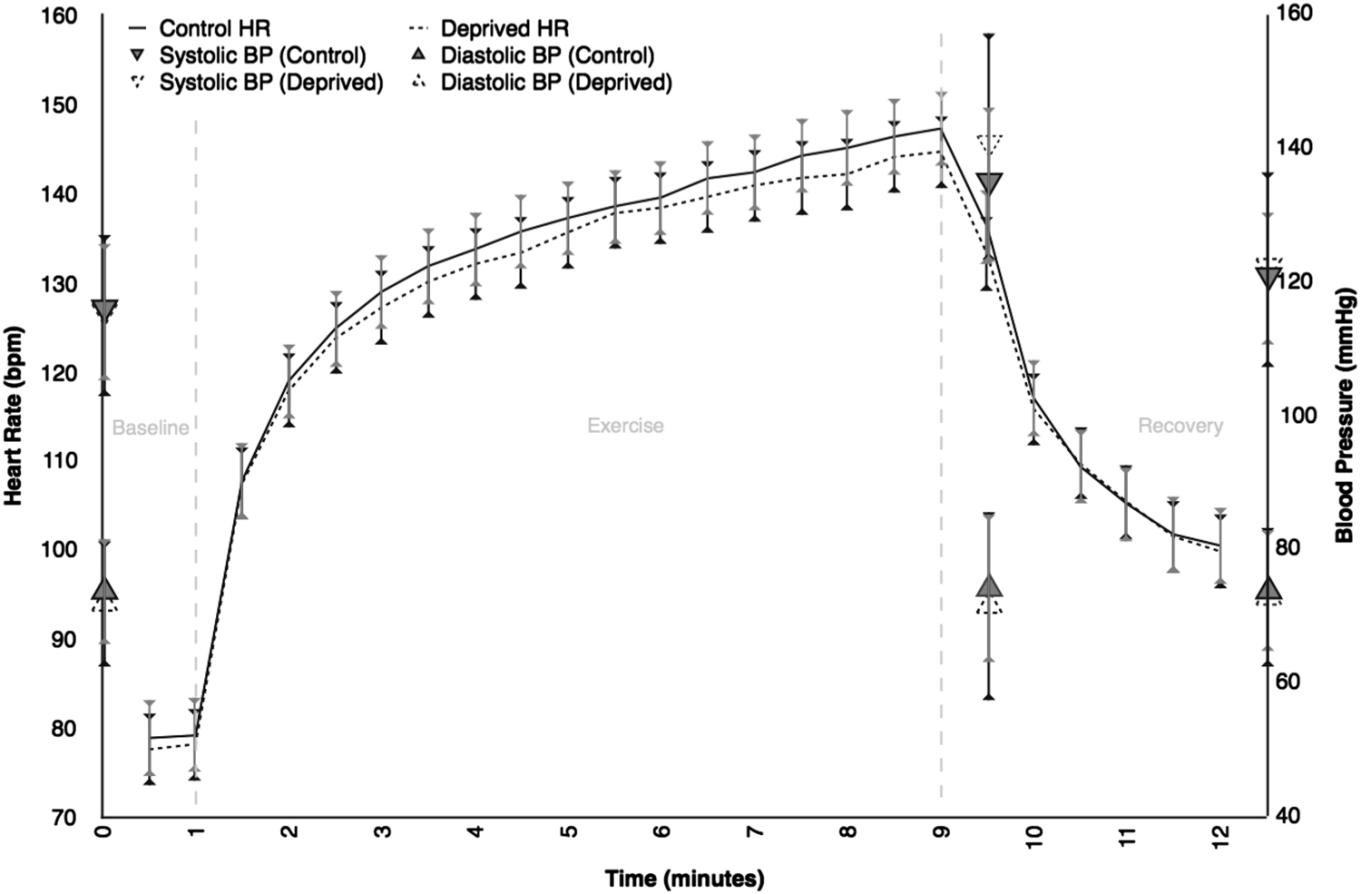
Line graph comparing heart rate during the baseline, exercise, and recovery periods of cardiopulmonary exercise testing between the control and deprived groups (*n* = 54). *Error bars* indicate two standard deviations. MANOVA shows no significant difference in heart rate (*p* = 0.723). Average values for blood pressure as measured at rest, post-exercise, and post-recovery are also displayed. Post-exercise systolic BP was found to be significantly different (*p* = 0.012)

Fifty-four (95%) percent of participants completed both exercise tests. Table 2 shows key variables from the exercise test. There were no significant differences between the normal sleep and sleep deprived arms, except for systolic blood pressure post-exercise (135 ± 12 vs. 140 ± 17 mmHg; *p* = 0.012). There is no significant difference between the changes in mean heart rate and RPE when compared using a MANOVA test *p* = 0.723 and *p* = 0.559, respectively (see Fig. 3).

**Table 2:** Participant characteristics during CPET

Test	Normal night sleep (*n* = 54)	Sleep deprived (*n* = 54)	Difference	*p* value
Heart rate (bpm)				
Max heart rate	149 ± 22	146 ± 20	3 ± 14	0.079
Blood pressure (mmHg)				
Systolic at rest	116 ± 10	115 ± 12	1 ± 12	0.733
Mean arterial pressure at rest	88 ± 7	86 ± 9	2 ± 7	0.123
Systolic post-exercise	135 ± 12	140 ± 17	−6 ± 17	0.012*
Mean arterial pressure post-exercise	94 ± 7	94 ± 11	0 ± 11	0.812
Systolic post-recovery	120 ± 10	122 ± 15	−1 ± 13	0.429
Mean arterial pressure post-recovery	89 ± 7	89 ± 10	0 ± 9	0.908

Data are reported as mean ± standard deviation. Data analysed using paired two-tailed *T* test

*Indicates significant result at an alpha of 5% (*p* ≤ 0.05)

### Cognitive and physical testing

Table 3 compares the results of the cognitive tests and additional physical tests under sleep deprivation and normal sleep test conditions. There was no significant difference observed across all tests except reaction time, with a significantly higher average (*p* = 0.03) among individuals who were sleep deprived.

**Table 3:** Participant characteristics for cognitive tests

Test	Normal night sleep (*n* = 57)	Sleep deprived (*n* = 57)	Difference	*p* value
Memory				
Mean sequence length	10 ± 4	10 ± 3	0 ± 3	0.307
Stroop				
Monochrome				
Time (s)	37 ± 6	38 ± 6	−1 ± 1	0.185
Errors (*n*)	1 ± 1	2 ± 1	0 ± 0	0.268
Conflicting colors				
Time (s)	40 ± 7	41 ± 8	−1 ± 1	0.123
Errors (*n*)	1 ± 1	1 ± 1	0 ± 0	0.768
Color block				
Time (s)	53 ± 10	52 ± 10	0 ± 0	0.437
Errors (*n*)	1 ± 2	1 ± 1	0 ± 0	0.409
Conflicting words				
Time (s)	73 ± 17	72 ± 16	1 ± 1	0.469
Errors (*n*)	2 ± 3	2 ± 3	0 ± 0	0.866
Reaction time				
Mean (s)	0.18 ± 0.04	0.19 ± 0.03	−0.15 ± 0.04	0.030*
Spirometry				
FEV_1_ (L)	3.63 ± 0.99	3.67 ± 0.95	−0.03 ± 0.25	0.303
FVC (L)	4.33 ± 1.19	4.36 ± 1.22	−0.03 ± 0.19	0.321
FEV_1_/FVC	0.85 ± 0.09	0.85 ± 0.08	−0.01 ± 0.06	0.511
PEFR (L/min)	467 ± 134	468 ± 137	−1 ± 79	0.930

Data are reported as mean ± standard deviation. Data analysed using paired two-tailed *T* test

*Indicates significant result at an alpha of 5% (*p* ≤ 0.05)

The results of the first visit and second visit were compared irrespective of condition for the cognitive tests and reaction time. There was a significant difference for the monochrome, color blocks, and conflicting words Stroop tests (*p* = 0.001, *p* = 0.000, and *p* = 0.011, respectively); no significant difference was found in other variables.

## Discussion

The main findings of this study were that sleep deprivation resulted in a significant increase in reaction time and post-exercise systolic BP in university students after one night of sleep deprivation, compared to a normal night’s sleep.

### Cognitive

No significant differences were found in the cognitive tests, suggesting that one night of sleep deprivation has minimal effect on a student’s cognitive capacity.

Working memory and executive function both heavily rely on the prefrontal cortex, anterior cingulate cortex, and salience network. This network has been shown reduced activation post-acute sleep deprivation [31–35]. Interestingly, a comprehensive review has noted that particular tests show no significant difference, such as the digit span test. This test is most similar to the assessment of memory used in our study [35, 36]. Another study found that both partial and total sleep deprivation had no effect on visual working memory, but that total sleep deprivation had a significant effect on filtering efficiency [37]. As the Simon© game tests visual working memory, our results suggest that the effect of sleep deprivation may not be as widespread in university students, with components of memory being preserved [31].

The stroop test showed no significant increase in time taken to complete, or the number of mistakes made in each set. Acute sleep deprivation has been demonstrated to have no effect on the principal processes of interference or facilitation with performance influenced by increased reaction time [15]. Previously, the stroop test has been found to show practice effects [20]. This was evident in the present study with significant improvements made on the second visit, irrespective of participant condition. While it is difficult to interpret the reason for this result, it has previously been demonstrated that with increased age, there is an increase in brain activity in response to acute sleep deprivation [38]. Therefore, the young student population may be more effective at dealing with acute sleep deprivation.

Working memory and executive function are important with regard to university students, since they are linked to the understanding of complex concepts. Indeed, the previous research has shown a stronger correlation between attainment and working memory than with Intelligence Quotient [39]. Our study indicates that acute sleep deprivation was not detrimental to students’ cognitive ability; however, a review of other cognitive variables is necessary [35].

### Physical

This study found an increase in reaction time after sleep deprivation, which has previously been well described with student subjects [40]. The underlying physiology of this effect is localised to the anterior cingulate cortex, middle prefrontal gyrus, and inferior parietal lobes which have been shown to be hypoactivated in acute sleep deprivation. This finding is pertinent to students, in particular the large proportion of them (52%) who take part in sport at least once a week [7, 31, 41]. Of greater concern, the previous research has demonstrated that tired students are more likely to drive dangerously, another activity requiring prompt reactions. In one study of 1039 students, 16% reported falling asleep while driving [42]. Another found that an ‘all-nighter’, had a comparable effect on driving performance to a blood alcohol concentration of 0.1% which is above the UK Drink Driving limit [43]. Reaction time has also been linked with cognitive processing speed via mental chronometry, indicating that the effect of slowed reaction time may not be wholly in the realm of physical exertion, but also a pseudo measurement of IQ [44].

RPE is commonly described as involving aspects of both metabolism and CNS activity [45, 46]. There was no significant change in RPE in the present study, although mean RPE after sleep deprivation was higher than after a night of sleep. This contradicts much of the pre-existing literature, which suggests that sleep deprivation is associated with a significantly increased RPE but little or no change in physiological parameters [35, 47].

This study found a reduction in HR post-exercise after acute sleep deprivation, although not significantly. A previous study has shown following two nights of sleep deprivation, and HR was significantly lower, during, and after an exercise test, compared to two nights of sleep. This was speculated to be due to ACTH concentration, which was lowest on the second day of sleep deprivation [48]. This is the same period over which exercise testing was carried out in our study, and could explain the effect on HR found.

According to the present study, acute sleep deprivation had no effect on resting blood pressure, but caused an increase in systolic blood pressure post-exercise. Thus, the effect on blood pressure from this study was unclear, a finding which has been reflected in the literature [49, 50]. Sleep deprivation has previously been demonstrated to stimulate sympathetic activity and neuroendocrine response to stressor stimuli [47]. Therefore, continuous periods of sleep deprivation, e.g., before a project deadline, may lead to students developing hypertension or having inappropriate response to intense exercise.

Spirometry was used to assess lung function parameters including FVC, FEV_1_, and the FEV_1_/FVC ratio. Spirometry is commonly used in clinical practice for monitoring baseline lung function. Analyses showed no statistically significant difference when comparing the sleep deprived values against the values for the normal night’s sleep, a finding supported by the literature [51, 52].

## Limitations

Several limitations need to be considered when interpreting the findings of this study. First, participants carried out their night of sleep deprivation in an environment of their choice rather than a supervised environment. Therefore, the study design was reliant on self-reported sleep deprivation and form completion, which may mean that some students had more sleep than others on the sleep deprivation night. Whilst this reduces the generalisability of our results in more diverse samples, the results are indicative of the effects of acute sleep deprivation on students in higher education. Alternate equipment and a larger test selection would have given a wider holistic prospective on the impact of sleep deprivation in university students; however, the interventions used in the study were chosen to maximise participant familiarity and minimise testing time.

## Conclusion

This study found that acute sleep deprivation has a significant effect on postexercise blood pressure and reaction time in students. These changes are likely due to neuroendocrine changes and downregulation in salience and motor areas of the brain. Most notably reduced reaction times will impact competitive sports, and can pose a danger to safety critical actions such as driving. However cognitive and neurophysical impairment in other functions was not as widespread as previously thought. Overall, this study found that an “all-nighter” does not affect a student’s cognitive ability, whilst physical performance is significantly affected.
